# Ischemic Stroke and Savings in Time to Achieve Functional Recovery: Experience from NeuroAiD

**DOI:** 10.3390/jcdd10030117

**Published:** 2023-03-12

**Authors:** Narayanaswamy Venketasubramanian, Yogesh Pokharkar, Jia Hui Chai, Christopher Li Hsian Chen

**Affiliations:** 1 Raffles Neuroscience Centre, Raffles Hospital, Singapore 188770, Singapore; 2 Singapore Clinical Research Institute, Consortium for Clinical Research and Innovation Singapore, 23 Rochester Park, Singapore 139234, Singapore; 3 Memory Aging and Cognition Centre, Department of Pharmacology, Yong Loo Lin School of Medicine, National University of Singapore, Singapore 117600, Singapore

**Keywords:** MLC601, NeuroAiD, functional recovery, stroke, time to recovery

## Abstract

Despite recent progress with revascularisation interventions after acute ischemic stroke, many patients remain disabled after stroke. Using data from a multi-centre, randomised, double-blind, placebo-controlled trial of a neuro-repair treatment (NeuroAiD/MLC601) with a long-term follow-up, we analysed the savings in time to functional recovery, measured by a modified Rankin Scale (mRS) score of 0 or 1, in patients receiving a 3-month oral course of MLC601. Analysis of time to recovery was assessed by a log-rank test and hazard ratios (HRs) adjusted for prognosis factors. A total of 548 patients with baseline NIHSS scores 8–14, mRS scores ≥ 2 at day 10 post-stroke, and at least one mRS assessment on or after month 1 were included in the analysis (placebo = 261; MLC601 = 287). Time to functional recovery was significantly shortened for patients receiving MLC601 versus patients receiving placebo (log-rank test: *p* = 0.039). This result was confirmed by Cox regression adjusting for the main baseline prognostic factors (HR: 1.30 [0.99, 1.70]; *p* = 0.059) and was more pronounced in patients with additional poor prognosis factors. The Kaplan–Meier plot showed that approximately 40% cumulative incidence of functional recovery was achieved within 6 months after stroke onset in the MLC601 group versus 24 months in the placebo group. The main findings are that MLC601 reduced the time to achieve functional recovery, and a 40% functional recovery rate was achieved 18 months earlier compared to placebo.

## 1. Introduction

Stroke is a leading cause of death and disability worldwide, and its burden continues to increase particularly in low- and middle-income countries [1]. Thus, the primary and secondary prevention of strokes is essential, combining healthy lifestyle recommendations, treatments of cardiovascular risk factors (CVRFs) and secondary prevention interventions [2,3]. Unfortunately, these recommendations often fail for economic and social reasons.

In parallel with revascularisation, intensive research has been carried out on different therapies aimed at protecting brain cells in the penumbra area surrounding the infarct core during ischemia, and where the initially viable tissue is at risk of delayed cell death [4]. For several decades, many potential neuroprotectants have been tested, showing promising effects in preclinical models, but to date, none have demonstrated a significant long-term clinical effect on post-stroke functional recovery [5].

The final stage of post-stroke recovery mechanisms is neurorepair, combining several processes initiated spontaneously by the brain including axon sprouting and preservation, dendritic branching, neurogenesis, remyelination, blood–brain barrier integrity, and development of new brain cortical maps and networks [6]. Thrombolysis and thrombectomy changed stroke care in the 1990s and 2010s, respectively. Over the next decade, increased neural repair and recovery after stroke could revolutionise the management and treatment of these patients [6]. Unlike other organs, the central nervous system (CNS) has a limited capacity for self-repair and helping to overcome this limitation would transform the way we might treat patients with post-stroke neurological impairments [7].

MLC601 is a multi-ingredient formulation derived from traditional Chinese medicine, administered three times a day in capsules containing extracts from raw herbal and non-herbal components, and developed and marketed by Moleac Pte (MLC) under the name NeuroAiD^TM^ [8]. Its neuroreparative properties have been well established in animal and cellular models of global and focal cerebral ischemia, traumatic injuries, neuroinflammation and neurodegeneration [9,10,11,12,13]. Based on data from a placebo-controlled study of a neurorepair treatment (NeuroAiD/MLC601) with long-term follow-up [14,15], the analysis presented here aims to characterize and quantify a specific clinical benefit of NeuroAiD, namely the time saved as compared to placebo to achieve functional recovery (i.e., modified Rankin Scale (mRS) score 0 or 1) in patients who received a 3-month oral course of MLC601 after acute ischemic stroke (AIS).

## 2. Materials and Methods

### 2.1. Study Design

Data from the CHInese Medicine Neuroaid Efficacy on Stroke recovery (CHIMES) study and its double-blind follow-up extension (CHIMES-E) were used for this analysis [14,15]. This was an international, multi-centre, randomised, double-blind study comparing a 3-month course of NeuroAiD (MLC601) to placebo in terms of post-stroke recovery assessed with mRS at baseline, day 10, and months 1, 3, 6, 12, 18 and 24. MLC601 is a multi-ingredient formulation derived from traditional Chinese medicine by Moleac Pte. (MLC) and marketed under the trade name NeuroAiD^TM^. Study treatment was administered three times a day in capsules containing extracts from raw herbal and non-herbal components [14], in addition to standard stroke care including control of vascular risk factors, anti-thrombotics and appropriate rehabilitation. The two studies were approved by the respective institutional review boards of the study sites, and written informed consent was gathered from all subjects or legal representatives [14,15].

### 2.2. Study Population

A total of 1099 subjects with an AIS of mild to moderate severity, with baseline of the National Institutes of Health Stroke Scale (b-NIHSS) scores from 6 to 14, were randomised in the CHIMES study. Stroke severity was determined at baseline using the 15-item NIHSS, gathered within 72 h of stroke onset. mRS was used to assess functional recovery at all follow-up time points from discharge or day 10 to month 24. The population considered for this exploratory analysis includes all subjects with b-NIHSS 8–14, as those with b-NIHSS 6–7 had limited symptoms and tended to recover spontaneously: by month 3, 68% of these patients had recovered in this subgroup, while only 32% in the rest of the CHIMES population (b-NIHSS 8–14) had recovered. Additionally, patients should have an mRS ≥ 2 at discharge or day 10 and at least one post-baseline mRS assessment (i.e., at least one assessment on or after month 1). The purpose of these other inclusion criteria was to exclude patients without confirmed impairment or having no mRS assessment after day 10 (or discharge) to calculate time to functional recovery.

### 2.3. Statistical Analysis

The primary outcome for the proposed aim was the time to achieve functional recovery (mRS 0–1), measured in months from baseline to first visit/assessment when the patient achieved mRS 0 or 1. The lost-to-follow-up patients in the selected population were censored at the last available non-missing mRS assessment in the analysis of time to functional recovery. The descriptive summary of baseline variables was provided by treatment arms to assess the comparability of baseline characteristics between the two treatment arms.

The time to functional recovery was presented using a Kaplan–Meier plot of the cumulative incidence of functional recovery (mRS 0–1) for each treatment arm. The comparison between the two treatment arms (MLC601 vs. placebo) was performed using a log-rank test. The difference in functional recovery rates, 95% confidence interval (CI) and *p*-value between the treatment arms were derived with the normal approximation method and the standard errors for the difference were computed with the Greenwood formula [16].

Furthermore, the adjusted hazard ratio (HR) of MLC601 vs. placebo, 95% CI and *p*-value were estimated using a Cox proportional hazards model adjusted for age (≤60 years vs. >60 years), time from stroke onset to study treatment (≤48 h vs. >48 h) and mRS score at day 10. The unadjusted hazard ratio, its 95% CI, and *p*-value were also presented as supporting evidence. The model aims to compare the outcome between the treatment groups conditioned on baseline covariates to balance the confounding effect due to the covariates even if they were imbalanced at baseline.

Subgroup analyses were performed for the prognostic factors [17,18], which are time from stroke onset to study treatment (OTT > 48 h), baseline NIHSS total score (10 to 14), and rehabilitation (Yes) during the first 3 months, by using Kaplan–Meier analysis and the Cox proportional hazards model, including factors for treatment, subgroup and treatment by subgroup interaction.

Statistical analyses were performed using SAS version 9.4.

## 3. Results

### 3.1. Baseline Characteristics

**Figure 1:**
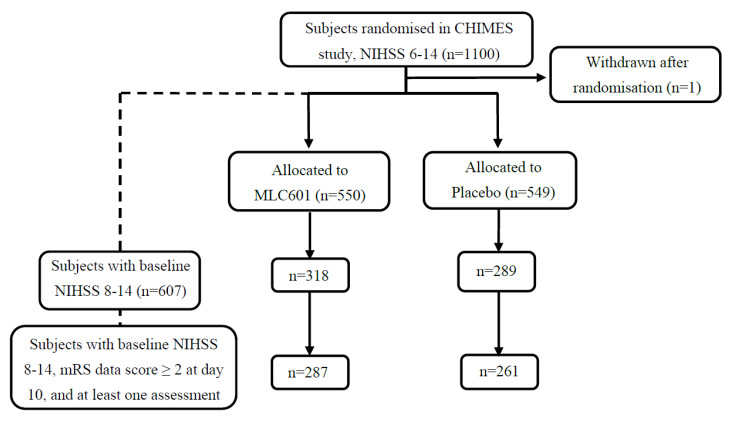
Flow chart of study population included in analysis of time savings.

As shown in Figure 1, 548 patients with b-NIHSS scores between 8 and 14, and mRS scores ≥ 2 at day 10, and which had at least one mRS assessment on or after month 1 were included in the analysis (261 in placebo arm and 287 in MLC601 arm). Baseline characteristics were comparable and well balanced between two treatment groups (Table 1). The overall mean age and its standard deviation (SD) was 62.2 (11.2) years, and 59.5% of the participants were males. The median (Q1, Q3) b-NIHSS total score (15 items) was 10.0 (9.0, 12.0). History of vascular events and cardiovascular risk factors were also well balanced between study groups.

### 3.2. Savings in Time to Functional Recovery

**Figure 2:**
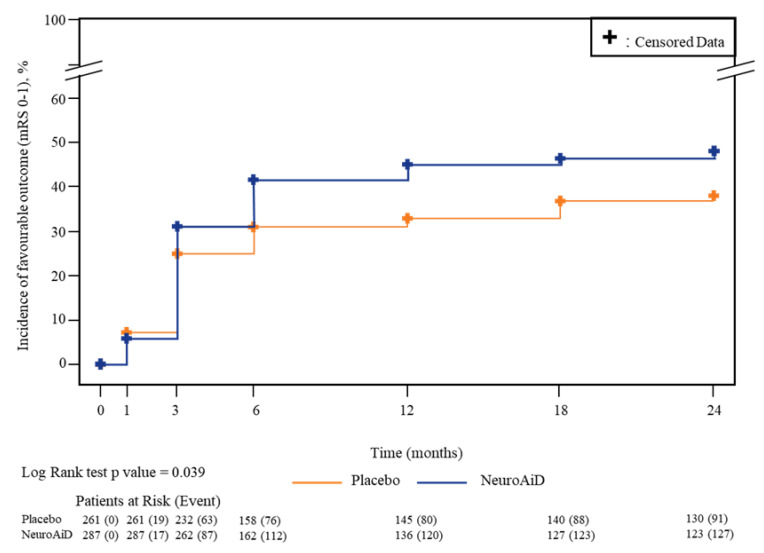
Kaplan–Meier plot for cumulative incidence of patients that achieved functional recovery (mRS 0–1).

The results in Figure 2 show that faster functional recovery (mRS 0–1) is achieved in the MLC601 group compared to placebo (log-rank *p* = 0.039). The cumulative incidence of functional recovery (mRS 0–1) was significantly higher (Table 2) in the MLC601 group compared to placebo at each of the assessment time points from 6 to 24 months (42% vs. 31% at month 6 (*p* = 0.013), 45% vs. 33% at month 12 (*p* = 0.005), 46% vs. 37% at month 18 (*p* = 0.030) and 48% vs. 38% at month 24 (*p* = 0.027).

A cumulative incidence of functional recovery of approximately 38% was observed in the placebo group at month 24, while a similar rate (42%) was achieved in the MLC601 group at month 6, which is 18 months earlier compared to placebo (Table 2). As shown in Table 2, this result was confirmed by a Cox proportional hazard model adjusted for the potential baseline prognostic covariates (adjusted HR: 1.30; 95% CI: 0.99 to 1.70; *p* = 0.059) and without adjustment (HR: 1.35; 95% CI: 1.02 to 1.75; *p* = 0.035).

The time to achieve functional recovery was significantly shorter in the MLC601 group compared to the placebo group in all three subgroups, i.e., NIHSS score of 10–14 at baseline (HR: 1.53, 95% CI: 1.04 to 2.24), OTT > 48 h (HR:1.72, 95% CI: 1.15 to 2.59), and rehabilitation during the first 3 months (HR:1.60, 95% CI: 1.05 to 2.43). Furthermore, the cumulative incidence of functional recovery at 24 months was significantly higher in the MLC601 group compared to the placebo arm in all three subgroups (Table 3).

### 3.3. Safety Analysis

The safety of MLC601 was reported in detail in the CHIMES publication, showing no differences in terms of adverse events (AEs) and serious AEs (SAEs) between the MLC601 and placebo groups at 3 months [14], and without differences in significant medical events over 24 months in the CHIMES-E study [15]. A safety quantitative analysis in patients with b-NIHSS 8–14 showed that there was no difference between MLC601 and placebo in the proportions of patients with AEs (41% in both groups) or SAEs (11.5% vs. 12.3%, respectively). No subject discontinued the study due to an AE.

## 4. Discussion

This exploratory analysis conducted in a population of patients that had an AIS of intermediate severity (NIHSS 8–14 at baseline) shows that time to achieve functional recovery (mRS 0–1) was significantly shortened in MLC601-treated patients compared to placebo. Functional recovery rates are significantly higher in patients treated with MLC601 compared to those treated with placebo at all assessment time points from 6 to 24 months. To achieve a similar functional recovery rate of approximately 40%, patients on placebo need about 24 months, while those on MLC601 will only need 6 months. This represents a clinically significant time saving of approximately 18 months and a potential gain in terms of health benefit, as well as savings of time and healthcare resource utilisation. The adjusted HR and unadjusted HRs results both showed trends in the same direction across the study population. This supports a favourable effect of MLC601 on savings in time to functional recovery, since the HRs are consistent and measured with good precision.

The savings in time to achieve a favourable outcome are confirmed in patients with more severe AIS (NIHSS 10–14 at baseline) or who had poor prognostic factors such as delayed hospitalisation (48–72 h after stroke onset) or who needed rehabilitation. The significantly higher value of the HR (1.72) in this subgroup (time from stroke onset to treatment >48 h) represents a 63% [=1.72/(1 + 1.72)] higher chance for a patient in the MLC601 group to achieve functional recovery earlier as compared to a patient in the placebo group. The clinical efficacy of MLC601 in patients whose treatment was initiated after 48 h or in those who experienced a more severe stroke (b-NIHSS 10–14) could be due to the exclusion of patients who may have had transient ischemic attacks or other underdiagnosed conditions, thus increasing the analysed proportion of patients with an impairment due to stroke. For patients who had a rehabilitation program during the first 3 months, the time savings are probably related to the fact that MLC601 has shown neuroreparative properties in in vitro studies and animal models [8] by stimulating neuroplasticity and neurogenesis, thus contributing to an optimal recovery of brain functions, as already shown in previous studies [17,18].

As explained in the Methods section, we excluded patients with a b-NIHSS of 6 or 7 at baseline from the present analysis, as spontaneous recovery rates at month 3 were high (approximately 68%) and similar in both groups, placebo and MLC601. A meta-analysis published in 2019 compared the effects of thrombectomy at month 3 to a control group receiving medical treatment [19]. From the selection of 2484 records, this meta-analysis included 5 studies in the final analysis with a total of 733 cases, of which 226 were composed of 113 matched pairs, with b-NIHSS cut-offs of ≤5 or ≤8, and regardless of rt-PA use. In all these subgroup analyses, the meta-analysis did not find a correlation between endovascular treatment and good or favourable clinical outcomes in terms of efficacy and complications (SIH). The authors conclude that their results should be confirmed by further high-quality trials. These results support our decision to exclude patients with a b-NIHSS score of 6 or 7 from our analysis, who are between the two thresholds ≤5 and ≤8, and to select patients with b-NIHSS 8–14 for the analysis of time savings.

The choice of mRS 0–1 as a criterion for evaluating functional recovery in the CHIMES study and in the present analysis of time savings was based on the fact that the scores 0 and 1 correspond homogeneously to the absence of significant disability, with the patient being able to perform all pre-stroke activities. On the other hand, an mRS score of 2 qualifies patients with a slight disability, as they are unable to perform all pre-stroke activities, which introduces some heterogeneity when using mRS 0–2 as an efficacy criterion instead of mRS 0–1. Additionally, a major meta-analysis of nine intravenous thrombolysis (IVT) trials published in 2014 used mRS 0–1 as the efficacy endpoint, which was considered a good stroke outcome with no significant disability at 3 and 6 months [20].

Other clinical studies showed a benefit of NeuroAiD (MLC601, MLC901) in terms of time savings. A previous study of MLC601 combined with rehabilitation after an ischemic stroke compared to rehabilitation alone in the placebo arm showed that the benefits of MLC601 are more evident earlier and for longer in subjects receiving persistent rehabilitation, supporting a combined time-saving effect of MLC601 with rehabilitation [21]. In a pilot clinical trial of MLC901 in patients recruited within 12 months of a mild traumatic brain injury (TBI), the main endpoints analysed were the trajectories over time for the cognitive domains of complex attention and executive functioning, as they are the most commonly affected areas of cognitive functioning after TBI [22]. Improvements in complex attention were cumulative over time, while they remained relatively stable in the placebo group, and if an improvement of executive functioning was observed in both groups, it was accelerated in the MLC901 group.

Thanks to its multi-ingredient formulation, MLC601 has a multi-target mode of action [8]. The improvement in post-stroke recovery with MLC601 treatment is most likely due to the synergistic effect of its nine herbal extracts and their active compounds, each of which may target a different mechanism. This led to the development of a new formulation, MLC901 (NeuroAiD^TM^II), containing only the nine plant extracts that were found to have the same pharmacological properties as MLC601 [8], with favourable results in terms of post-treatment recovery in patients that experienced stroke and TBI [9,13]. The nine herbs are Radix astragali, Radix salvia miltiorrhizae, Radix paeoniae rubra, Rhizoma chuanxiong, Radix angelicae sinensis, Carthamus tinctorius, Prunus persica, Radix polygalae and Rhizoma acori tatarinowii [8,12]. This combination of plant extracts releases many active ingredients, including astragaloside IV (AST-IV), salvianolic acid B (SAB), tanshinone IIB (TSB), tetramethylpyrazine (TMP), ferulic acid, β-asarone, yellow safflower hydroxyl A (HSYA), total peony glycoside (TPG) and presenegenin [9,12]. Thus, MLC601 and MLC901 reduce infarct size and ischemia-induced neurological deficits, attenuate cerebral ischemia-induced pro-inflammatory cellular infiltration, and attenuate stroke-induced increased expression of pro -inflammatory mediators (IL11, IL1β, IL6 and TNFα) in the brain [12]. Overall, by using a combination of multiple herbal ingredients, with both the herbal extract or the active ingredient, MLC601 enables a multi-pathway targeting approach, while a single targeted drug treatment can disrupt the homeostasis of the human body system and potentially exert serious side effects. It can be hypothesized that the combined effects of the multi-herb formulation on several pathways involved in stroke progression could explain the significantly faster post-stroke recovery responsible for the time savings.

The question of diagnostic delay remains an issue for revascularization procedures, in particular for IVT, the benefits of which diminish quite rapidly over time. A retrospective cohort study confirmed the importance of reperfusion rate with modern endovascular techniques and reviewed the use of CT without perfusion as a diagnostic technique [23]. Even if an early diagnosis of AIS is desirable for all patients, the margin of delay is much greater for the initiation of a neuroreparative treatment such as MLC601, as confirmed by the analysis of the time savings in the patients included between 48 and 72 h after the stroke onset.

While strokes are largely preventable, as shown by the reduction in incidence rates worldwide, stroke remains the third leading cause of death and disability combined worldwide [1]. Thus, millions of stroke victims with an impairment can benefit from a treatment, such as MLC601, allowing savings in time to functional recovery in patients after a stroke of intermediate severity. The benefits that can be expected from savings in time to achieve functional recovery after a stroke concern the patients, their family and society. As shown in a recent analysis carried out with MLC601 [24], a quicker recovery limits the risk of complications and serious adverse events related to disability, and reduces the duration of hospitalisation. Another analysis supporting a combined effect of MLC601 with rehabilitation showed that addition of MLC601 reduced the time spent in rehabilitation compared to rehabilitation alone [21]. All these time savings should reduce associated costs. Finally, a return to a normal life and work will undoubtedly be one of the greatest benefits for patients and their families.

The present analysis was an exploratory analysis and the trials from which the analysed data are extracted were not originally planned to assess time savings. As the vast majority of patients included in the analysis were of Asian ethnicity, caution is required when applying the findings to patients of other ethnicities, but the positive effects of MLC601 and MLC901, its simplified formulation, have been established in other populations outside of South-East Asia in stroke and other brain impairments [22,25,26,27]. Another limitation is that the location and volume of ischemic lesions observed in CT scans or MRIs were not captured in the CHIMES database.

The main strength of this new analysis is the data used in this analysis was from CHIMES and CHIMES-E, a well-conducted multicentre acute stroke trial with a large sample size and performed in a double-blinded, placebo-controlled manner, with a long-term follow-up. The investigators were experienced in stroke treatment, and the data collected was robust and unbiased to meet the objective of the new analysis. Blinding of the patients and investigators to treatment allocation was maintained throughout the 24 months, reducing assessment bias.

As regards further research, we suggest employing the proposed methodology used in this study with a longer treatment duration. It will also be helpful to test it in patients of other ethnicities. Studies of the possible cost savings associated with these reductions in time to achieve functional recovery have been planned.

## 5. Conclusions

The results of this analysis show that MLC601 (NeuroAiD) reduced the time to achieve functional recovery assessed by mRS compared to placebo. A functional recovery rate of 40% after a stroke was achieved within 6 months in the MLC601 group, while it took 18 more months to achieve the same rate in the placebo group, representing a clinically significant time saving. This favourable outcome is even more pronounced in patients with additional factors of poor prognosis in terms of stroke severity, need for rehabilitation and time to treatment access. We plan to conduct further research to analyse how these time savings translate into healthcare resource savings.

## Figures and Tables

**Table 1:** Baseline characteristics in overall analysis population.

Characteristics	Overall Analysed Population (b-NIHSS 8–14)		
	MLC601	Placebo	*p*-Value
N	287	261	0.44
Age (years), mean (SD)	61.4 (11.0)	63.1 (11.4)	0.08
Age > 60 years	145 (50.5)	155 (59.4)	
Gender, male, n (%)	166 (57.8)	160 (61.3)	0.43
OTT: Time from stroke onset to study treatment (hours), mean (SD)	49.8 (17.3)	50.0 (17.4)	0.89
Time from stroke onset to study treatment > 48 h, n (%)	148 (51.6)	145 (55.6)	
b-NIHSS total score (15 items), median (Q1, Q3)	10.0 (9.0, 12.0)	10.0 (9.0, 12.0)	0.68
mRS score at day 10, median (Q1, Q3)	4.0 (3.0, 4.0)	4.0 (3.0, 4.0)	0.90
Rehabilitation, n (%)	123 (42.9)	121 (46.4)	0.44
Vascular history and risk factors, n (%)			
Ischaemic cerebrovascular disease	25 (8.7)	21 (8.0)	0.88
Ischaemic cardiovascular disease	11 (3.8)	14 (5.4)	0.42
Peripheral vascular disease	3 (1.0)	1 (0.4)	0.63
Hypertension	238 (82.9)	212 (81.2)	0.66
Diabetes	82 (28.6)	77 (29.5)	0.85
Hyperlipidaemia	114 (39.7)	105 (40.2)	0.93
Smoking	124 (43.2)	108 (41.4)	0.73
Habitual alcohol intake	77 (26.8)	62 (23.8)	0.43

b-NIHSS: baseline NIH stroke scale; SD: Standard deviation; mRS: modified Rankin Scale. To compare the two treatments groups (MLC601 vs. Placebo), *p*-values were obtained using the two-sample *t*-test for continuous variables, Fisher’s exact test for categorical variables, and the Wilcoxon rank-sum test for discrete variables.

**Table 2:** Time to achieve functional recovery (mRS 0–1): Functional recovery rates and hazard ratio (MLC601 vs. Placebo) in the whole study population.

	Placebo (N = 261)	MLC601 (N = 287)	*p*-Value
Functional recovery (mRS 0–1), n (%)	91 (34.9)	127 (44.3)	
Censored, n (%)	170 (65.1)	160 (55.7)	
**Time to functional recovery (months)**			
**At 3 months ^#^**			
Functional recovery rate (95% CI)	24.9 (19.7, 30.3)	31.1 (25.7, 36.5)	
Difference in rates (95% CI)	-	6.2 (−1.4, 13.8)	0.111
**At 6 months ^#^**			
Functional recovery rate (95% CI)	31.0 (25.3, 37.0)	41.7 (35.7, 47.6)	
Difference in rates (95% CI)	-	10.7 (2.3, 19.0)	0.013
**At 12 months ^#^**			
Functional recovery rate (95% CI)	32.9 (27.0, 39.0)	45.1 (38.9, 51.1)	
Difference in rates (95% CI)	-	12.2 (3.6, 20.7)	0.005
**At 18 months ^#^**			
Functional recovery rate (95% CI)	36.8 (30.6, 43.0)	46.4 (40.2, 52.4)	
Difference in rates (95% CI)	-	9.6 (0.9, 18.4)	0.030
**At 24 months ^#^**			
Functional recovery rate (95% CI)	38.2 (32.0, 44.5)	48.2 (41.9, 54.2)	
Difference in rates (95% CI)	-	9.9 (1.1, 18.7)	0.027
Hazard ratio (95% CI) ^$^	-	1.35 (1.02, 1.75)	0.035
Adjusted hazard ratio (95% CI) ^&^	-	1.30 (0.99, 1.70)	0.059

# Functional recovery rates (95% CI) were obtained from a Kaplan–Meier curve; the difference in rates (95% CI) and *p*-value are derived using the normal approximation method and standard errors are computed with the Greenwood formula. &: Obtained from Cox regression model adjusted for age (<=60 years vs. >60 years), time from stroke onset to study treatment (<=48 h vs. >48 h) and mRS score at day 10. $: Obtained from Cox regression without any covariate adjustment. NIHSS: NIH Stroke Scale; mRS: modified Rankin Scale, CI: Confidence interval.

**Table 3:** Time to achieve functional recovery over 2 years in subgroups with poor prognostic factors: Functional recovery (mRS 0–1) rate at month 24 and hazard ratio (MLC601 vs. placebo).

Subgroups with Poor Prognostic Factors	Placebo	MLC601	*p*-Value
**Baseline NIHSS 10–14**	153	175	
Functional recovery rate (95% CI) ^#^	30.3 (22.7, 38.1)	42.9 (35.0, 50.5)	
Difference in rates (95% CI)	-	12.6 (1.6, 23.6)	0.025
Hazard ratio (95% CI) ^&^	-	1.53 (1.04, 2.24)	0.030
**Time from stroke onset to treatment > 48 h**	145	148	
Functional recovery rate (95% CI) ^#^	29.2 (21.5, 37.2)	44.9 (35.9, 53.4)	
Difference in rates (95% CI)	-	15.7 (3.9, 27.5)	0.009
Hazard ratio (95% CI) ^&^	-	1.72 (1.15, 2.59)	0.009
**Rehabilitation during first 3 months**	121	123	
Functional recovery rate (95% CI) ^#^	32.9 (24.3, 41.7)	48.0 (38.4, 57.0)	
Difference in rates (95% CI)	-	15.1 (2.3, 28.0)	0.021
Hazard ratio (95% CI) ^&^	-	1.60 (1.05, 2.43)	0.027

#: Event rates (95% CI) are obtained from a Kaplan–Meier curve; difference in rates (95% CI) and *p*-value are derived using the normal approximation method and standard errors are computed with the Greenwood formula. &: Obtained from Cox regression model adjusted for treatment (MLC601 vs. Placebo), subgroup and treatment by subgroup interaction. NIHSS: NIH Stroke Scale; mRS: modified Rankin Scale; CI: Confidence interval.

## Data Availability

All data generated or analysed during this study are included in this article, along with references to data from cited published studies. The database is not publicly available. Further enquiries can be directed to the corresponding author, Narayanaswamy Venketasubramanian.
